# Clinical evaluation of an authorized medical equipment based on high performance liquid chromatography for measurement of serum voriconazole concentration

**DOI:** 10.1186/s40780-021-00225-8

**Published:** 2021-11-09

**Authors:** Kazutaka Oda, Shota Uchino, Kayo Kurogi, Mai Horikawa, Naoya Matsumoto, Kou Yonemaru, Hitomi Arakaki, Taiki Katsume, Kaho Matsuyama, Tomomi Katanoda, Yuki Narita, Koji Iwamura, Hirofumi Jono, Hideyuki Saito

**Affiliations:** 1 Department of Pharmacy, Kumamoto University Hospital, 1-1-1, Honjo, Chuo-ku, Kumamoto, Japan; 2 Department of Clinical Pharmaceutical Sciences, Graduate School of Pharmaceutical Sciences, Kumamoto University, 1-1-1, Honjo, Chuo-ku, Kumamoto, Japan

**Keywords:** Voriconazole, High performance liquid chromatography, Therapeutic drug monitoring

## Abstract

**Background:**

Therapeutic drug monitoring for voriconazole is recommended for its optimum pharmacotherapy. Although the feedback of the measurement result of serum voriconazole concentration by outsourcing needs a certain time (days within a 1 week), there was no medical equipment for the measurement available in clinical practice. Recently, a medical equipment based on high performance liquid chromatography, named LM1010, has been developed and authorized for clinical use. In this study, to validate the clinical performance of LM1010, we compared the measured serum voriconazole concentrations by LM1010 with those by outsourcing measurement using liquid chromatography-tandem mass spectrometry.

**Methods:**

We conducted the observational study approved by the institutional review board of Kumamoto University Hospital (No. 1786). Residual serum samples harvested for therapeutic drug monitoring were separated. Measured concentrations by LM1010 by the standard filter method (needs serum volume of > 400 μL) or the dilute method (needs serum volume of 150 μL) were compared with those by outsourcing, respectively. Acceptable measurement error range of 0.72–1.33 was considered. There were 69 serum samples, where the 35 or 34 samples were employed for evaluation of the standard filter method or the dilute method, respectively.

**Results:**

The measured concentration using the standard filter method/outsourcing was 2.22/2.10 μg/mL as the median, 1.57–3.40/1.53–3.62 as the interquartile range, < 0.2–10.76/< 0.2–11.46 μg/mL as the range, while those using the dilute method/outsourcing was 2.36/2.29 μg/mL as the median, 1.08–2.94/1.03–3.06 as the interquartile range, 0.24–10.00/< 0.2–10.85 μg/mL as the range. The regression line for the standard filter method or the dilute method were y = 0.935x + 0.154 or y = 0.933x + 0.162, respectively. The standard filter method or the dilute method showed 11.4% samples (4/35, 95%CI 3.2–26.7%) or 8.8% samples (3/34, 95%CI 1.9–23.7%) out of the acceptable measurement error range, respectively.

**Conclusion:**

Measurement of serum voriconazole concentration by LM1010 can be acceptable in clinical TDM practice.

**Supplementary Information:**

The online version contains supplementary material available at 10.1186/s40780-021-00225-8.

## Background

Voriconazole, an azole antifungal agent, plays a pivotal role in the treatment of invasive aspergillosis [1]. For its optimum pharmacotherapy, therapeutic drug monitoring (TDM) is highly recommended based on TDM guideline, since dose dependent antifungal activity and hepatotoxicity have been frequently observed [2]. Therapeutic range at trough concentration of voriconazole has been introduced to be approximately 1–2 μg/mL as the lower limit and 4–5 μg/mL as the upper limit at 5–7 days after first dose [2]. For the measurement of serum voriconazole concentration, high performance liquid chromatography (HPLC) has been employed in clinical settings [3], although conventional HPLC needs special skills and plenty of time that can disturb usual clinical practice. Therefore, serum voriconazole concentration is inevitably measured by outsourcing, in which liquid chromatography-tandem mass spectrometry (LC-MS/MS) is used. However, it needs a certain time (days within a 1 week) to obtain the feedback of the measurement result, then opportunity for the dose optimization will come at more than 10 days after first dose at last. Because median days of therapy for development of voriconazole-induced hepatotoxicity is reportedly 10 days [4], the outsourcing measurement should be discouraged for maximal safe medication. It means that the measurement in individual hospitals should be encouraged. Nevertheless, none of medical equipment for the measurement of serum voriconazole concentration had been authorized in Japan, the situation can be of the serious concern in the treatment of invasive aspergillosis [1].

In 2020, a medical equipment for TDM was finally authorized in Japan (LM1010 High performance liquid chromatograph: LM1010, produced by HITACHI High-Tech Science, Tokyo), which can measure serum concentrations of 17 drugs including voriconazole based on HPLC. LM1010 automatically runs after pretreatment of samples (about 30 min required for 5 samples) and needs the least personal skills. Although the development for the measurement methods were reported [5], the clinical performance for each drug has yet to be evaluated. The aim of this study was to compare the measured serum voriconazole concentrations by LM1010 using HPLC with those by outsourcing measurement using LC-MS/MS.

## Main text

This observational study was conducted after approval from the institutional review board of Kumamoto University Hospital (No. 1786). Written informed consent was waived and the information was open in an opt-out manner using the document displayed in our website, where the opportunity to be withdrawn from this study were also provided. Blood serum samples were treated by centrifugation in 13,000 *g* × 4 min using serum separating medium followed by obtaining serum samples, which were originally used for measurement of voriconazole concentration by outsourcing in usual clinical TDM practice. Its residual serum samples of > 400 μL were separated from January 2020 to March 2021 for this study. These samples were immediately managed on the day of samplings, then stored at − 80 °C in a deep freezer until measurement, which was performed within next 9 months. Pretreatment of the separated residual samples for measurement was as follows: First, a standard filter method allows to filter residual samples of > 400 μL using a 1 mL syringe (TERUMO, Tokyo) connected with 0.45 μm syringe filter (HITACHI High-Tech Science, Tokyo) followed by obtaining filtered samples of more than 150 μL. A dilute method allows to dilute residual samples of 150 μL using equivalent volume of ultrapure water by following centrifuged in 13,000 *g* × 4 min followed by obtaining diluted samples of 300 μL. Second, solid phase extraction (SPE) using Spin column (HITACHI High-Tech Science, Tokyo) was employed as the sample purification (the detailed flow was provided in Supplementary Fig. S1), then the eluted sample was applied to an auto-sampler basically installed in LM1010 (the available validation data was provided in Supplementary Table S1). LM1010 allows voriconazole assay using a calibration curve developed by only one concentration, a method of HPLC with ultraviolet detection as the analytical method. The one measurement cycle is 7 min. We used the evaluation system for LM1010 (separating column: LaChrom LM Type A, integrated) in this study (the detailed measurement condition was unavailable). The measured concentrations were compared with those by outsourcing using LC-MS/MS (BML, Inc., Tokyo). The acceptable measure was considered that a 95% confidence interval (CI) of the corresponding regression line in the case within an acceptable measurement error range. Because a measurement error for one sample can be permitted within 15% (0.85–1.15) [6], comparison using two concentrations (by LM1010/outsourcing) can lead to the acceptable measurement error range of 0.72–1.33. Measurements using the dilute method were performed by the first author, while the measurements using the standard filter method were performed by other students (listed in the authors) of third grade in School of Pharmacy of Kumamoto University who were all unfamiliar with the operation of any chromatography systems.

**Fig. 1:**
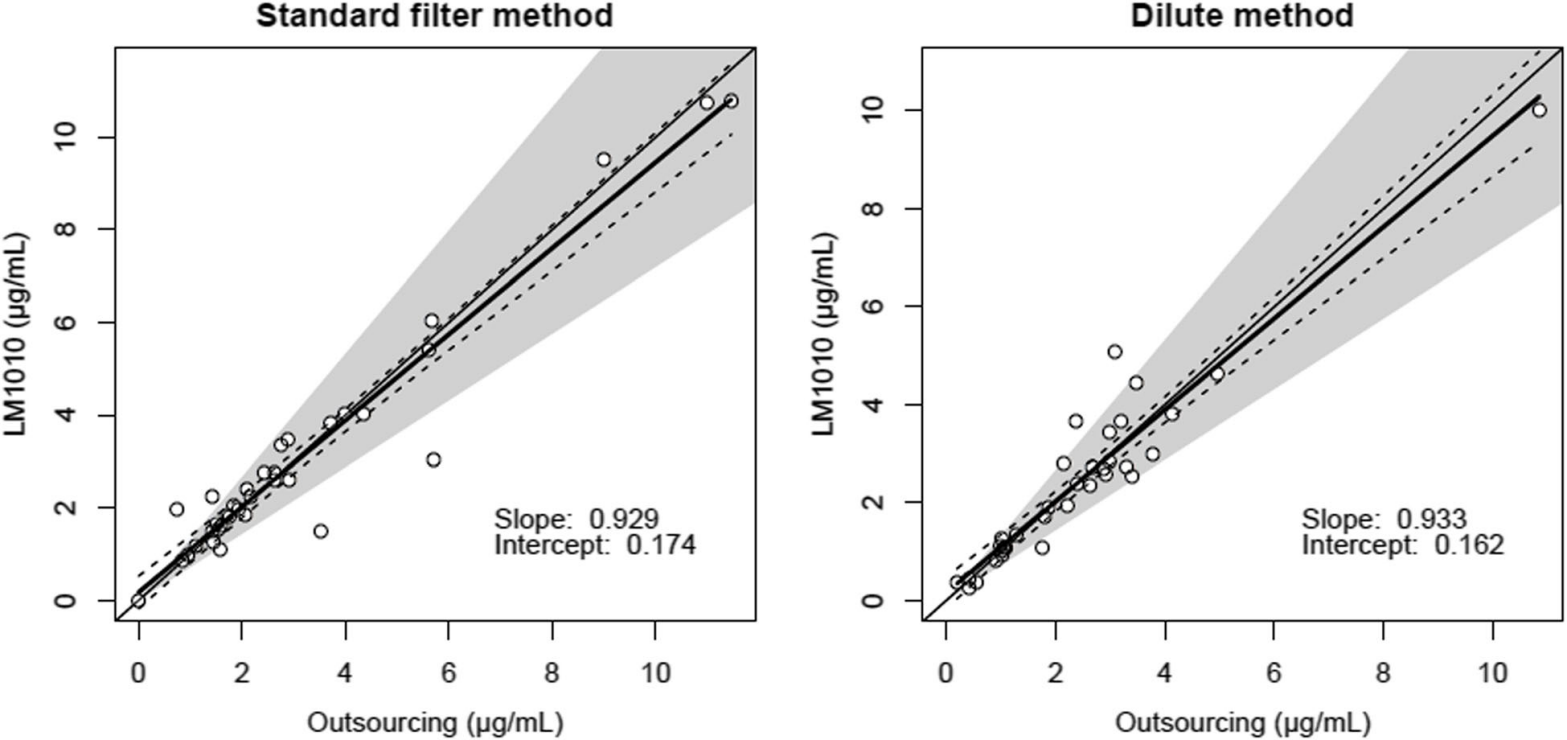
Regression analysis for measured concentrations by LM1010 against outsourcing. The left panel is of the standard filter method, while the right panel is of the dilute method. White circles indicated measured values. Bold lines indicate regression lines. Dashed lines indicate 95% CI of corresponding regression lines. Shade area indicate the acceptable measurement error range of 0.72–1.32

As the result, 69 serum samples were collected for the comparison, where the 35 or 34 samples were employed for evaluation of the standard filter method or the dilute method, respectively. The measured concentration using the standard filter method/outsourcing was 2.22/2.10 μg/mL as the median, 1.57–3.40/1.53–3.62 as the interquartile range, < 0.2–10.76/< 0.2–11.46 μg/mL as the range, while those using the dilute method/outsourcing was 2.36/2.29 μg/mL as the median, 1.08–2.94/1.03–3.06 as the interquartile range, 0.24–10.00/< 0.2–10.85 μg/mL as the range. The regression line for evaluations using the standard filter method or the dilute method were y = 0.935x + 0.154 or y = 0.933x + 0.162, as displayed in Fig. 1a and b, respectively. Both of the 95% CIs of the corresponding regression lines totally involved the line of y = x. Incidentally, 11.4% samples (4/35, 95%CI 3.2–26.7% based on binomial distribution) using the standard filter method or 8.8% samples (3/34, 95%CI 1.9–23.7% based on binomial distribution) using the dilute method were out of the acceptable measurement error range of 0.72–1.33. While systematic error was unobserved on the standard filter method, possible proportional error was observed on the dilute method based on Bland-Altman analysis (Supplementary Fig. S2a and b).

This study compared the measured serum voriconazole concentrations by LM1010 with those by outsourcing. As informed by the slopes of < 1.0 of the corresponding regression lines, the concentrations by LM1010 may be smaller than those by outsourcing. However, the small bias is likely to be permitted in clinical TDM practice. Previous studies provided the similar bias [7, 8]. Next, 7 concentrations out of acceptable measurement error range were observed in total, where 4 (2 in the standard filter method, 2 in the dilute method) concentrations were higher in LM1010 than outsourcing, remained 3 concentrations (2 in the standard filter method, 1 in the dilute method) were higher in outsourcing than LM1010. Although the former 4 concentrations might be affected by the specificity of the measurement system, all the uncontaminated target peaks were observed (data not shown). Common medication and trend in laboratory abnormalities were unidentified among the 4 patients. These facts can lower the possibility concerning specificity. Although the latter 3 lowered concentration using LM1010 might be attributed to degradation due to storage in a deep freezer, the association between degradation and storage durations was not associated (Supplementary Fig. S2c and d). LM1010 has been validated without an internal standard, which might be unassociated with the difference. Heterogeneous concentration might be generated by flicking mixing in the last step of sample preparation, where vortex could guarantee exact mixing. Both the concerned characteristics based on patients and the technique will be evaluated further. More importantly, inter-center variability could be concerned. Although information of the variability has been unavailable for voriconazole, that for immunosuppressants and antiepileptics have been available [9, 10]. The slopes for antiepileptics were variable such as 0.791–1.04 [9]. The coefficient variables (CVs%) for tacrolimus between different apparatuses was also variable such as 10.0–52.0% [10]. These findings appeared potential inter-center variability in any measurements, and the measurement error observed in this study was not atypical. Therefore, further evaluation should be performed to conclude the potential inter-center variability for LM1010 ideally in the same measurement system.

The standard filter method is originally recommended as the pretreatment in LM1010 [5], though sample volume of > 400 μL is necessary for the filtration to obtain samples of 150 μL. In some authorized medical equipment for TDM, concentrations are measured using samples of 200 μL. Sample volume of 400 μL in case of LM1010 may be excessive in the case of using residual samples harvested for other biochemical tests, or of neonates. Figure 1b indicated that the dilute method could address this concern without any specific items, though the observed possible proportional error should be further evaluated. Moreover, since students who has been unfamiliar with the operation of any chromatography systems provided the results by the standard filtration method, it was demonstrated that LM1010 requires no special skills. The measurement cost of LM1010 is variable depending on the license style but probably comparable with that of outsourcing (data not shown). Regarding the clinical meaning of the measurement in individual hospitals, the measurement result can rapidly be obtained as possible so that the rapidest pharmacotherapeutic optimization can be performed. Those points may contribute to preferable clinical outcomes such as maximized treatment efficacy and minimized adverse events, which in turn, may lead to possible medical cost reduction. The pharmacotherapeutic and pharmacoeconomic meanings in LM1010 should be evaluated in future.

## Conclusions

The measurement of serum voriconazole concentrations by LM1010, which is the only authorized medical equipment for measurement of serum voriconazole concentration, can be acceptable in clinical TDM practice even for samples with small volume.

## Supplementary Information

**Additional file 1: Fig. S1**. Sample preparation flow through solid phase extraction method. **Table S1**. Validation result for the measurement of voriconazole using LM1010. **Fig. S2**. Bland-Altman analysis for assessment of systematic errors on measurement methods.

## Data Availability

The datasets used and/or analyzed during the current study are available from the corresponding author upon reasonable request.

## Abbreviations

TDM: Therapeutic drug monitoring
HPLC: High performance liquid chromatography
LC-MS/MS: Liquid chromatography-tandem mass spectrometry

## References

[1] Ullmann AJ, Aguado JM, Arikan-Akdagli S, Denning DW, Groll AH, Lagrou K (2018). Diagnosis and management of aspergillus diseases: executive summary of the 2017 ESCMID-ECMM-ERS guideline. Clin Microbiol Infect.

[2] Matsumoto K, Takesue Y, Ohmagari N, Mochizuki T, Mikamo H, Seki M (2013). Practice guidelines for therapeutic drug monitoring of vancomycin: a consensus review of the Japanese Society of Chemotherapy and the Japanese Society of Therapeutic Drug Monitoring. J Infect Chemother.

[3] Yamada T, Mino Y, Yagi T, Naito T, Kawakami J (2012). Rapid simultaneous determination of voriconazole and its N-oxide in human plasma using an isocratic high-performance liquid chromatography method and its clinical application. Clin Biochem.

[4] Hanai Y, Matsuo K, Yokoo T, Ohtani M, Nishimura K, Kimura I (2015). Clinical course and risk factors for voriconazole-induced hepatotoxicity. Jpn J Pharm Health Care Sci.

[5] Morikawa G, Sorimachi M, Tamura K, Moriiwa Y, Shoji A, Okazawa K (2019). Development of a practical HPLC system for in-hospital analysis of blood concentration of various medicines. Bunseki Kagaku.

[6] U.S. Food % Drug Administration. Bioanalytical method validation guidance for industry. https://www.fda.gov/regulatory-information/search-fda-guidance-documents/bioanalytical-method-validation-guidance-industry, Accessed 1 Apr 2021.

[7] Badii P, Hashemizadeh Z, Montaseri H (2017). Therapeutic drug monitoring of Voriconazole: comparison of Bioassay with high-performance liquid chromatography. Jundishapur J Microbiol.

[8] Cattoir L, Fauvarque G, Degandt S, Ghys T, Verstraete AG, Stove V (2015). Therapeutic drug monitoring of voriconazole: validation of a novel ARK™ immunoassay and comparison with ultra-high performance liquid chromatography. Clin Chem Lab Med.

[9] Ikeya H, Harashima N, Kobayashi H, Ohyama M (2001). Interlaboratory difference in antiepileptic drug concentration and influence on TDM. Jpn J Med Tech.

[10] Miura M, Masuda S, Egawa Y, Yuzawa K, Matsubara K (2015). Interhospital laboratory variability and the imprecision of current immunoassay methods for tacrolimus from iMPT. Jpn J Transplant.

