# Supplementary material for: Clinical evaluation of an authorized medical equipment based on high performance liquid chromatography for measurement of serum voriconazole concentration
Source: J Pharm Health Care Sci. 2021 Nov 9;7:42. doi: 10.1186/s40780-021-00225-8 (PMC8576885; doi:10.1186/s40780-021-00225-8)
Supplement: Supplementary file 1 — Additional file 1: Fig. S1. Sample preparation flow through solid phase extraction method. Table S1. Validation result for the measurement of voriconazole using LM1010. Fig. S2. Bland-Altman analysis for assessment of systematic errors on measurement methods. [file 40780_2021_225_MOESM1_ESM.pptx]

## Slide 1
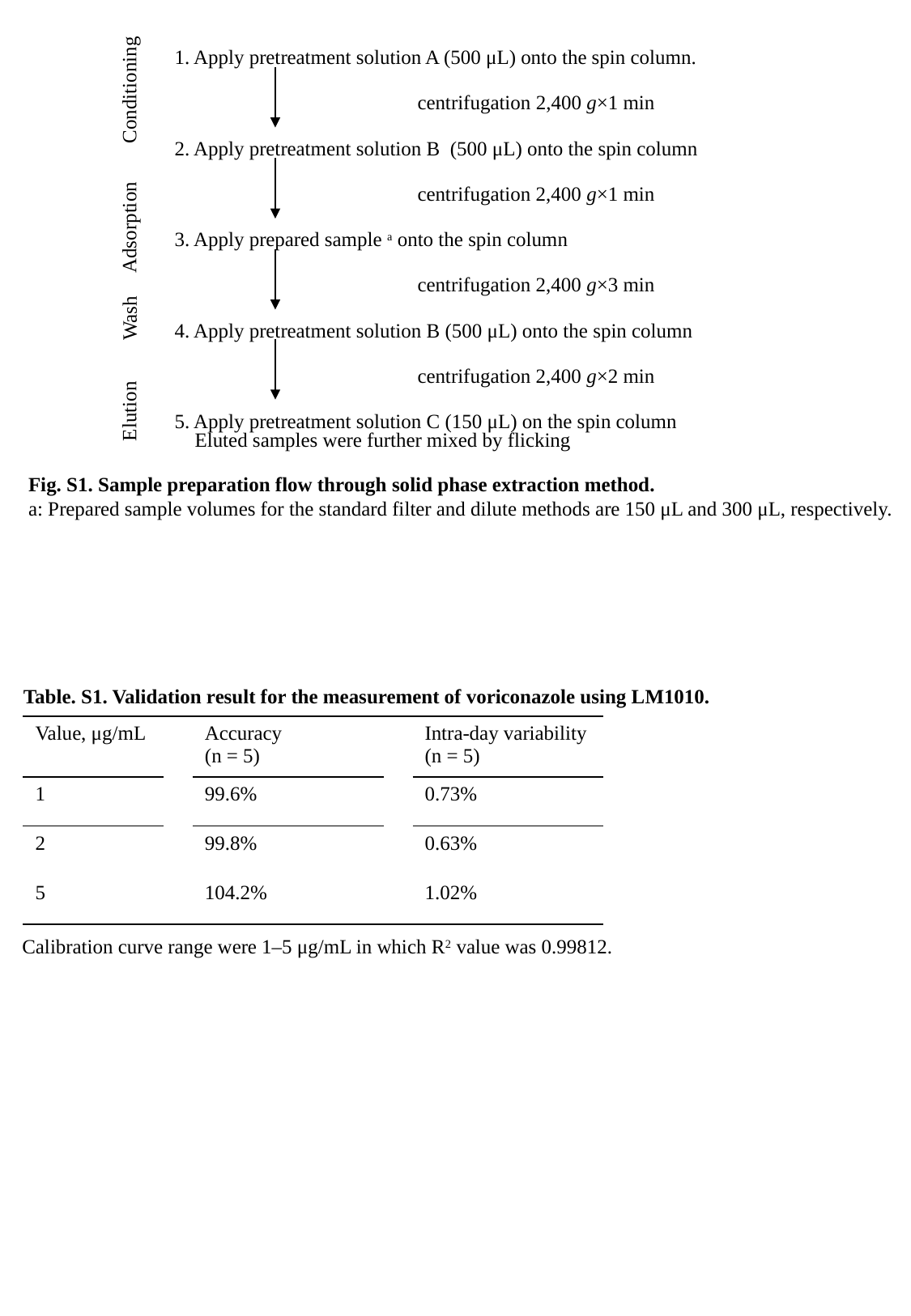

1. Apply pretreatment solution A (500 μL) onto the spin column.
		centrifugation 2,400 g×1 min
2. Apply pretreatment solution B (500 μL) onto the spin column
		centrifugation 2,400 g×1 min
3. Apply prepared sample a onto the spin column
		centrifugation 2,400 g×3 min
4. Apply pretreatment solution B (500 μL) onto the spin column
		centrifugation 2,400 g×2 min
5. Apply pretreatment solution C (150 μL) on the spin column
 Eluted samples were further mixed by flicking
Conditioning
Adsorption
Wash
Elution
Fig. S1. Sample preparation flow through solid phase extraction method.
a: Prepared sample volumes for the standard filter and dilute methods are 150 μL and 300 μL, respectively.
Table. S1. Validation result for the measurement of voriconazole using LM1010.
| Value, μg/mL | | Accuracy (n = 5) | | Intra-day variability (n = 5) |
| --- | --- | --- | --- | --- |
| 1 | | 99.6% | | 0.73% |
| 2 | | 99.8% | | 0.63% |
| 5 | | 104.2% | | 1.02% |
Calibration curve range were 1–5 μg/mL in which R2 value was 0.99812.

## Slide 2
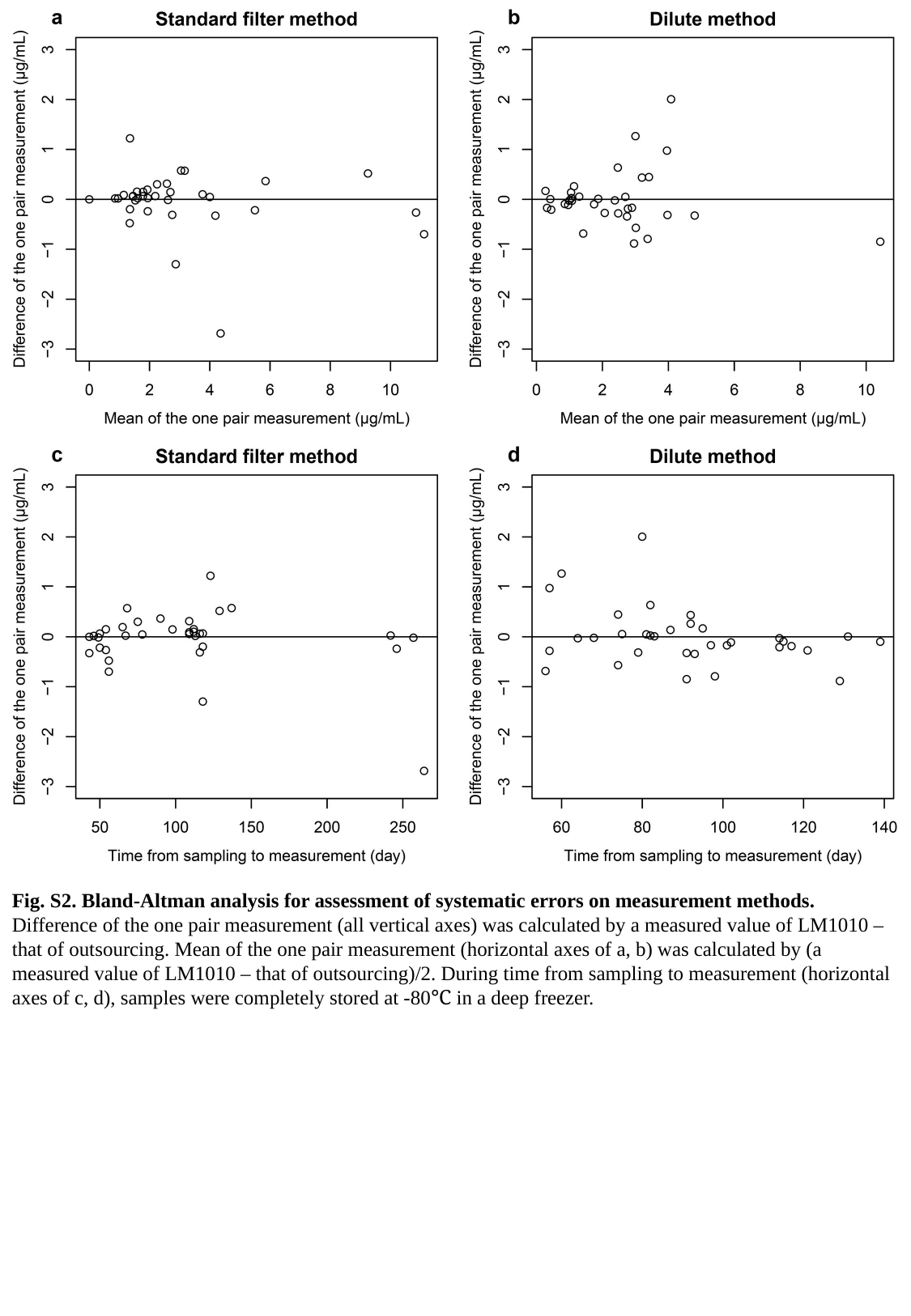

Fig. S2. Bland-Altman analysis for assessment of systematic errors on measurement methods.
Difference of the one pair measurement (all vertical axes) was calculated by a measured value of LM1010 – that of outsourcing. Mean of the one pair measurement (horizontal axes of a, b) was calculated by (a measured value of LM1010 – that of outsourcing)/2. During time from sampling to measurement (horizontal axes of c, d), samples were completely stored at -80℃ in a deep freezer.
